# Multiple leiomyosarcoma of great saphenous vein with lung metastasis

**DOI:** 10.1186/s13019-026-04368-3

**Published:** 2026-06-06

**Authors:** Shibo Zhao, Xiaofen Shang, Changqing Liu, Minghui Ou

**Affiliations:** Department of Vascular Surgery, Qingdao Municipal hospital, University of Health and Rehabilitation Sciences, Qingdao, 266000 Shandong People’s Republic of China

**Keywords:** Leiomyosarcoma, Saphenous vein, Lung metastasis, Multidisciplinary treatment

## Abstract

There are very few publications on unplanned excisions of great saphenous vein leiomyosarcomas (GSV-LMS). We describe a 64-year-old female patient with multiple leiomyosarcoma in the great saphenous vein (a total of 13 lesions) with lung metastasis. The patient was initially misdiagnosed and underwent a repeat surgical intervention. During the follow-up, a pulmonary nodule showed progressive enlargement, and the patient underwent pulmonary surgery. confirmed it to be a metastatic lesion. While some literature has documented this disease, the multidisciplinary treatment has not been emphasized, and the high incidence of lung metastasis has been overlooked. This is crucial importance of early identification and precise evaluation of lung metastasis. Given the paucity of cases, our case contributes valuable insights into the treatment approach necessary for optimal outcomes in GSV-LMS with lung metastasis. Multidisciplinary and aggressive treatment, under the guidance of the thoracic surgeon, remain paramount to improving survival in this rare malignancy with metastasis.

## Introduction

Leiomyosarcoma is a rare tumor that often leads to misdiagnosis and inappropriate treatment, with distant metastasis significantly increasing the risk of poor prognosis. The patient we report is a rare presentation of multiple leiomyosarcoma in the great saphenous vein (13 lesions in total) with lung metastasis. In this case, the misdiagnosis of this disease led to repeat surgery for the patient, as inadequate preoperative evaluation of pulmonary nodules resulted in unnecessary thoracic surgery. Although this case has been reported in the literature, it does not emphasize the presence of metastasis. As clinical experience shows, leiomyosarcoma is prone to hematogenous metastasis to the lungs, and for patients with a history of malignant tumors like leiomyosarcoma, newly detected pulmonary nodules are highly suspected to be metastatic. This awareness can be helpful in performing definitive surgery and avoiding multiple surgeries in similar complex cases. With the increase in cases report, such patients should no longer be managed solely by the vascular surgery department, instead, multidisciplinary management and comprehensive diagnosis and treatment should be considered.

## Case report

**Fig. 1:**
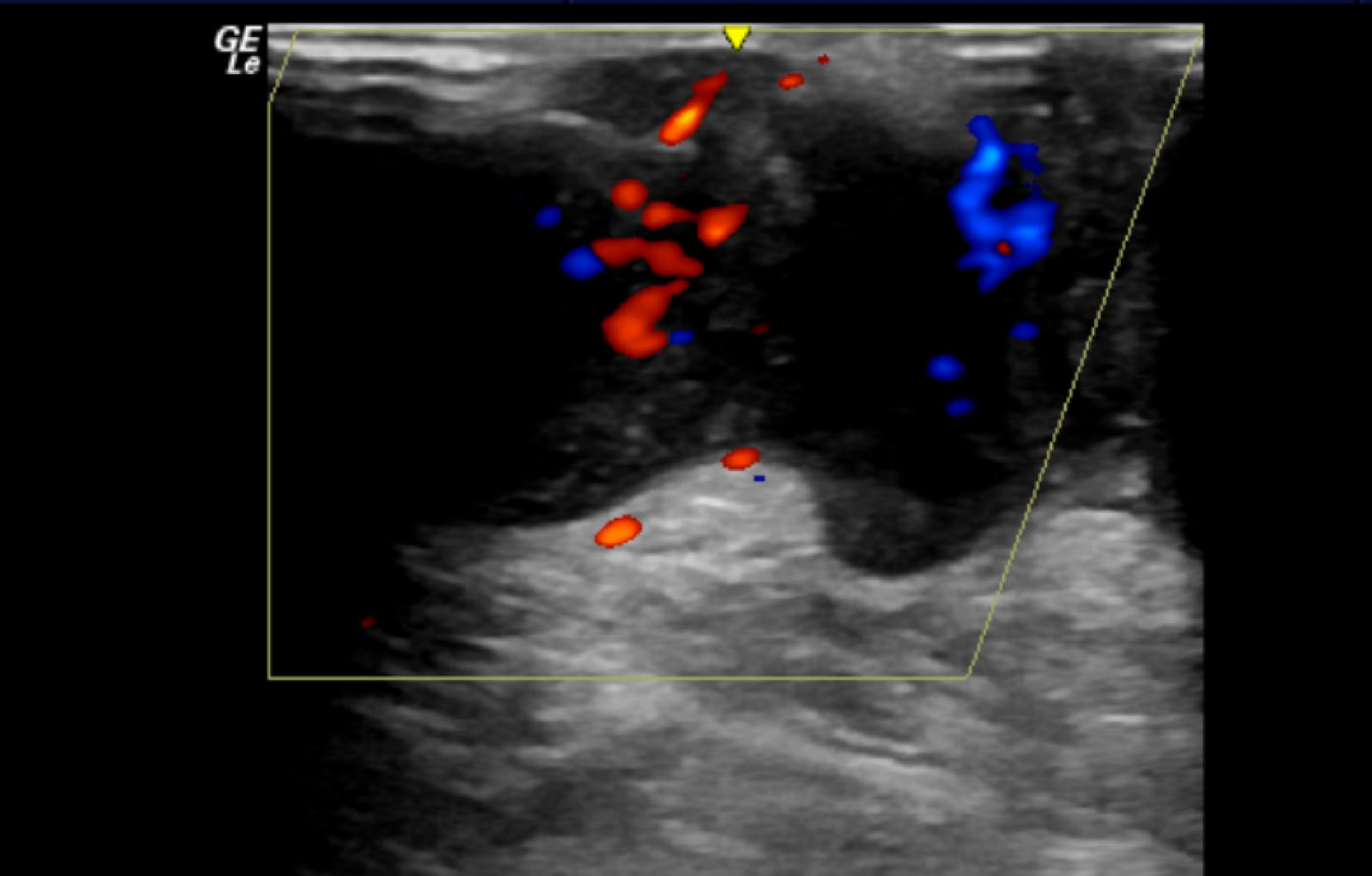
Ultrasound show intraluminal solid mass with both venous and arterial flow signals

**Fig. 2:**
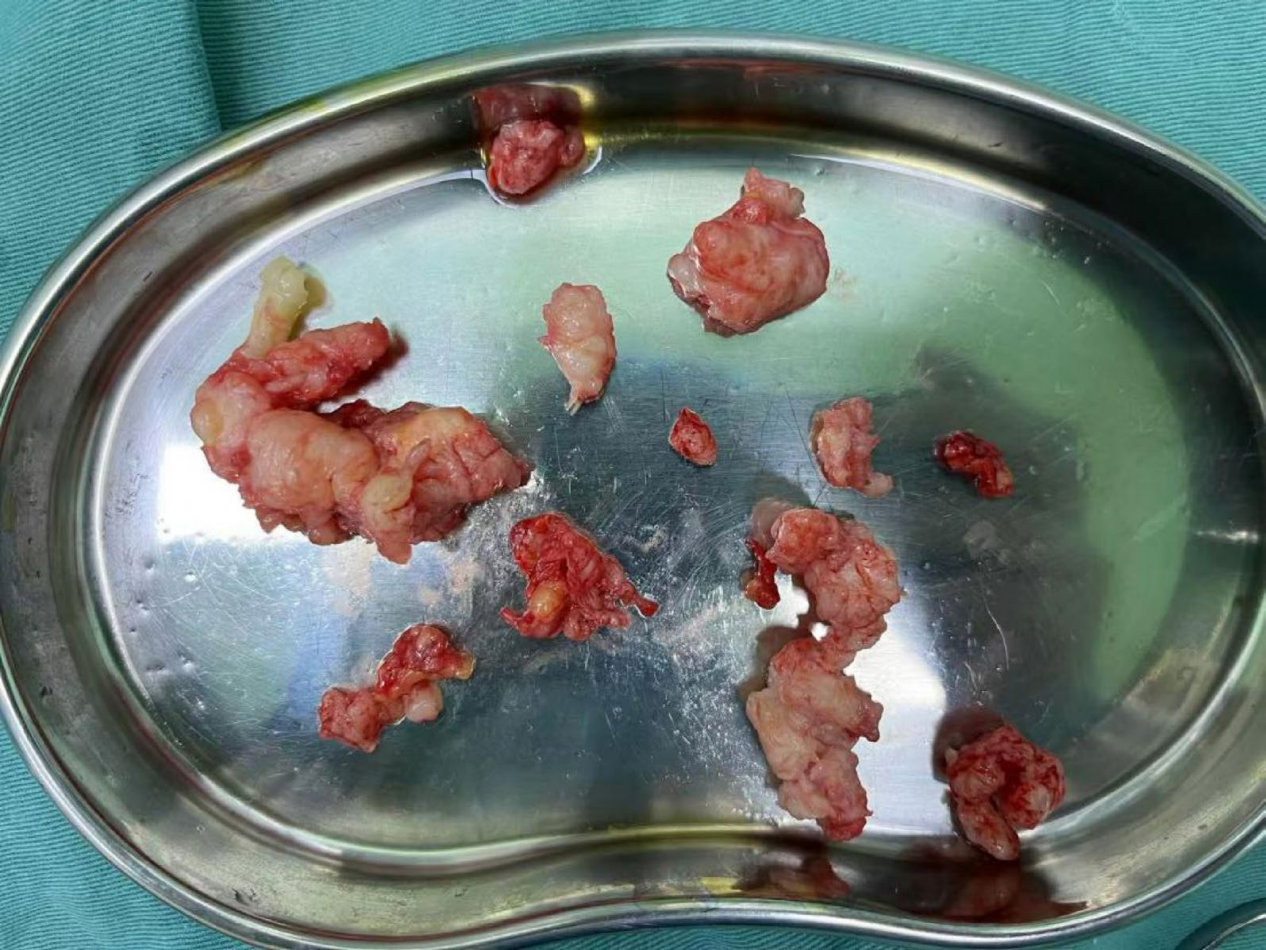
11 masses removed in the first operation, with diameters ranging from 5 mm to 60 mm

**Fig. 3:**
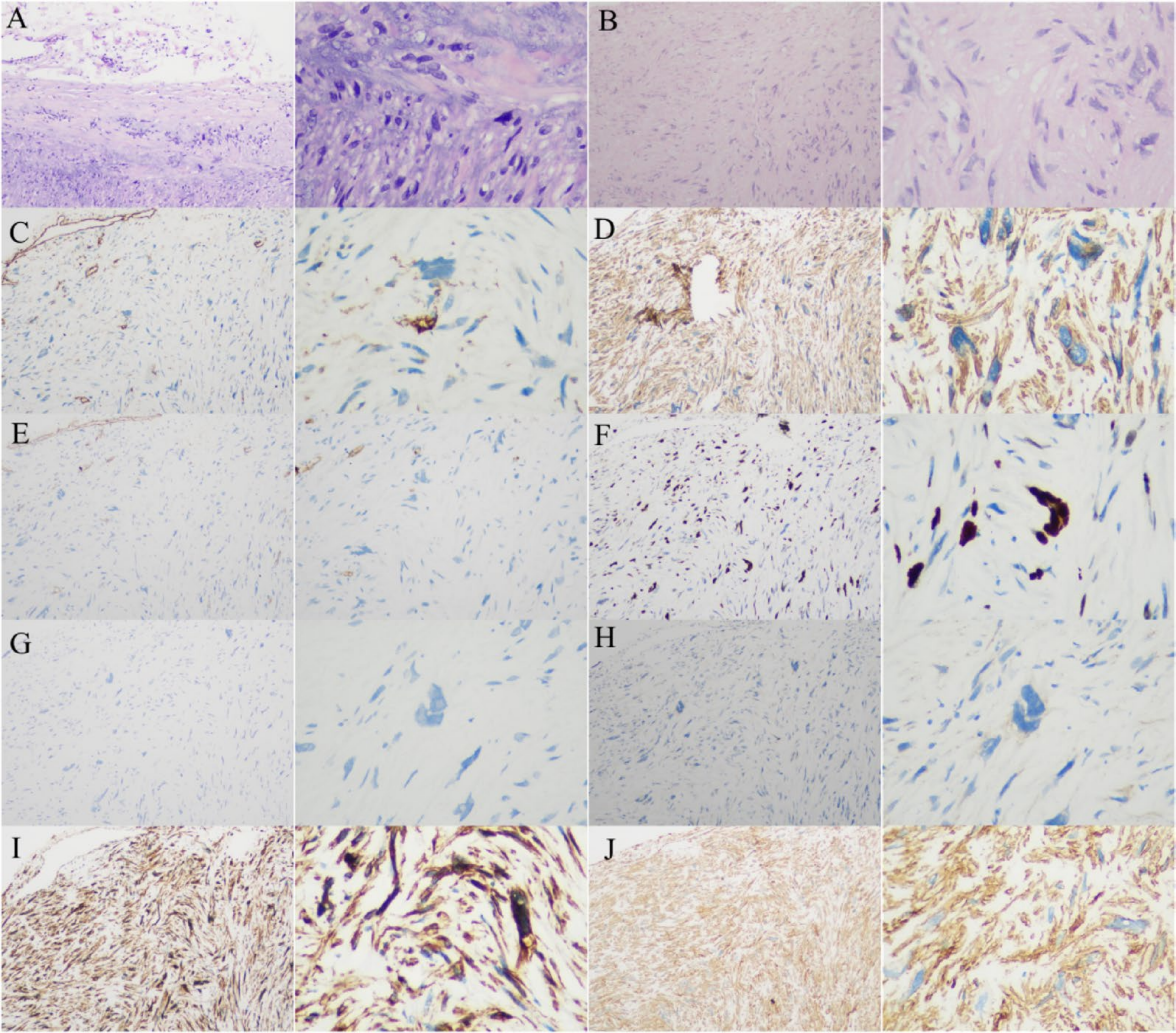
Immunohistochemistry: (**A**/**B**) H&E-stained pathological section of this case. It showed fusiform tumor cells with different nuclear sizes, irregular shapes, and large atypia, suggesting that the soft tissue pleomorphic tumor was more likely to be sarcoma. (**C**) IHC staining for CD31 shows positivity only in vascular structures. (**D**) IHC for h-caldesmon is partially positive. (**E**) IHC staining for CD34 shows positivity only in vascular structures. (**F**) IHC staining for Ki-67 is positive (30%), supported the diagnosis of high-grade leiomyosarcoma. (**G**) IHC staining for CK is negative. (**H**) IHC staining for S-100 is negative. (**I**) IHC staining for Desmin is partially positive. (**J**) IHC staining for SMA is partially positive. Left panel: (×100), Right panel: a locally magnified view (×400). IHC, immunohistochemistry

**Fig. 4:**
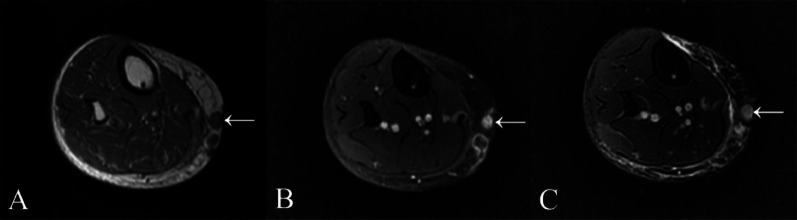
MRI: (**A**) Leiomyosarcoma appears hypointense on T1. (**B**) Hyperintensity on T1 + C. (**C**)T2 hyperintensity on fat-suppressed sequences. +C, contrast-enhanced

**Fig. 5:**
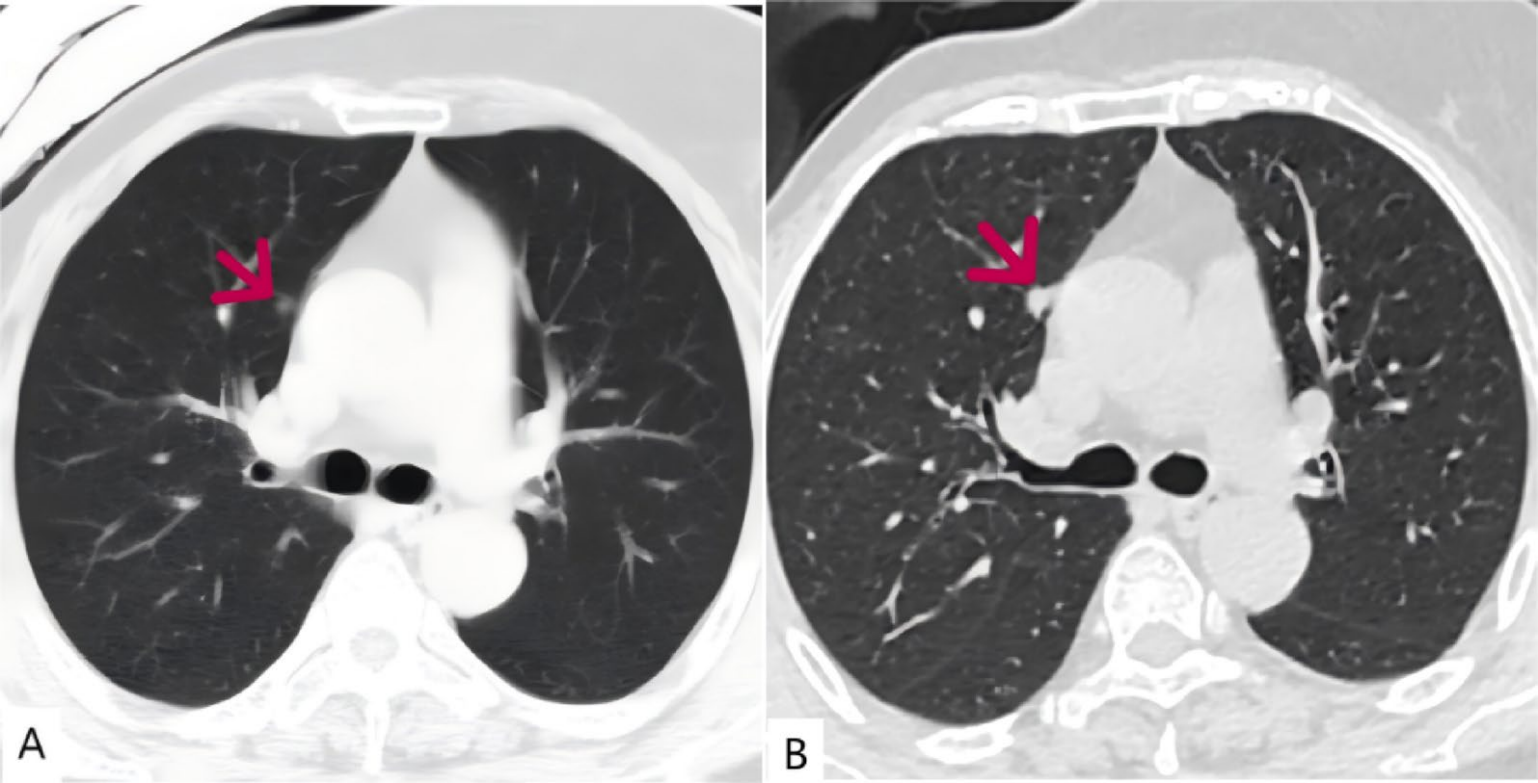
CT scan of pulmonary nodule: (**A**) initial nodule size 4 mm. (**B**) follow-up CT showed nodule 9 mm

An elderly female patient was admitted to our hospital in February 2023 for surgical management of right lower limb varicose veins with pain. Physical examination identified superficial varicose veins in the right lower limb and multiple painful indurations in the medial calf. Ultrasound examination of the lower extremity veins revealed varicose veins with localized thrombosis (Fig. 1). Preoperative lung CT showed multiple small ground glass nodules (up to 4 mm) in both lungs. According to the recommendation of the thoracic surgeon, the malignant transformation rate of 4 mm ground glass nodules is relatively low, and most are benign lesions. The patient had undergone radical mastectomy for right breast cancer in 1990. During surgery, routine procedures included high ligation of the great saphenous vein with laser closure. During calf vein stripping, grayish-white indurations adhered to the vessel walls were observed. A total of 11 irregular indurations were removed (Fig. 2) and sent for pathological examination, which confirmed leiomyosarcoma. Immunohistochemical results showed leiomyosarcoma (Fig. 3) (SMA (+), Desmin (+), CD117 (-), CD68 (-), CD163 (-), CD34 (vascular +), CD31 (vascular +), S100 (-), CK (-), Vimentin (+), Ki67 (≈ 30%+), β-catenin (+), SOX-10 (-), STAT6 (-), ERG (-), CR (-), H-cald (+)). Postoperative MRI (Fig. 4) and PET-CT revealed a patchy nodular shadow in the medial mid-calf subcutaneous tissue with increased FDG metabolism. Based on the examination results, the patient underwent a right lower limb soft tissue resection in April 2023. The excised specimen contained two grayish-white firm nodules, with pathology and immunohistochemistry indicating leiomyosarcoma. The Fe´de´ration Nationale des Centres de Lutte Contre le Cancer (FNCLCC) was G2. This patient was subsequently transferred to the oncology department for bed radiation therapy at a dose of 5500CGY over 25 sessions. Follow-up until June 2024 revealed multiple small nodules (maximum 9 mm) in both lungs, with some enlargement compared to previous findings (Fig. 5). A partial resection of the anterior segment of the right upper lobe and the basal segment of the right lower lobe was performed, and the resected pulmonary nodules were histopathologically confirmed as leiomyosarcoma. Postoperatively, this patient received AD chemotherapy regimen (doxorubicin+ dacarbazine) under the guidance of professional oncologists for 3 courses. To date, the patient has shown no recurrence or signs of distant metastasis during follow-up.

Detailed written informed consent was obtained from the patient for the publication of this case report and images.

## Discussion

According to the literature, since Aufrecht [1] first described a vascular leiomyosarcoma originating from the great saphenous vein in 1868, only about 57 cases have been reported to date. Most documented cases are solitary lesions with diameters ranging from 10 to 120 mm (mean approximately 41 mm) [2]. Due to its atypical clinical, owing to its presentation and nonspecific imaging features, it is often misdiagnosed as lipomas or schwannomas. In patients with lower limb varicose veins, it is particularly prone to being mistaken for superficial venous thrombosis. Among the reported cases, 43% experienced clinical misdiagnosis, resulting in delayed or inappropriate treatment.

Ultrasound remains the primary examination for evaluating superficial masses, yet its high misdiagnosis rate in vascular leiomyosarcoma demands diagnostic expertise shared by clinicians. In this case, the patient presenting with lower extremity varicose veins was initially misdiagnosed with venous thrombosis due to the ultrasound surgeon’s limited experience. The differential diagnosis involves distinct imaging features: thrombosed varicose veins typically show no blood flow signals, while partial recanalization may reveal venous flow patterns. Notably, this patient exhibited both venous and arterial flow signals with characteristic spectral patterns. MRI plays a pivotal role in diagnosing GSV-LMS [3, 4]. On T1WI, leiomyosarcoma appears isointense or slightly hypointense, while T2WI typically shows hyperintensity, particularly on fat-suppressed sequences. Clinical consensus suggests that biopsy may increase tumor dissemination and recurrence risks, making minimally invasive approaches crucial for prevention. For patients, avoiding invasive procedures carries significant clinical benefits [5].

Apart from focusing on the primary lesion, comprehensive examinations should exclude distant metastases, including potential sites such as thyroid, cardiac, scalp, skeletal, hepatic, cutaneous, cerebral, and pulmonary regions, with pulmonary metastasis being the most frequent [6, 7]. Cangiano et al. [7] hypothesized that over half of venous leiomyosarcoma patients may already have metastasis at diagnosis. A total of 57 cases have been reported in the literature, and our patient is the 58th documented case. Among these, 13 cases were confirmed to have distant metastasis, and 10 cases reported with lung metastasis (Table 1). However, early metastasis are relatively subtle, with small lesions that may be difficult to detect through routine examinations. Given the high rate of lung metastasis, thoracic surgeons need to pay particular attention to the condition of pulmonary nodules [8–12]. The possibility of metastasis should be suspected when pulmonary nodules are present in patient with GSV-LSM, particularly when the nodules do not meet surgical criteria. If feasible, a PET-CT should be further performed to comprehensively assess the presence of distant metastases.

Regarding the treatment of GSV-LMS, the current optimal treatment strategy appears to be wide resection of the tumor combined with local radiotherapy to reduce the risk of recurrence [7, 13]. Re-excision is the first option to be considered for the patient with unplanned suboptimal surgery. Previous case reports suggested unclear indications and efficacy of adjuvant chemoradiotherapy. However, the patients who achieving favorable outcomes with simple surgical resection had single and localized tumors, and short follow-up periods limit the assessment of long-term prognosis. For patients diagnosed with metastasis, the recommended treatment paotocol includes local resection, regional radiotherapy, and chemotherapy [14]. Most of the reported cases with distant metastasis showed poor prognosis [5, 15–21]. Megan H et al. [22] analyzed data from 18 randomized controlled trials, demonstrating that adjuvant chemotherapy reduced overall recurrence risk by 10% compared to local resection alone while improving overall survival by 6%. Pervaiz N et al. [23] found more significant adjuvant chemotherapy benefits in limb sarcomas through a meta-analysis. A total of 17 cases received adjuvant treatment after surgery, and none of these cases had local recurrence or metastasis during the reported postoperative follow-up period [24]. As more cases are reported and follow-up periods are extend, this prompts us to reconsider whether neoadjuvant or adjuvant chemotherapy is necessary to prevent distant metastasis or mitigate adverse prognostic risks. The Spanish Group for Research in Sarcomas (GEIS) [25] guidelines for soft tissue sarcoma recommend adjuvant chemotherapy for tumors located in the limbs, with neoadjuvant chemotherapy being the preferred option. The guidelines recommend neoadjuvant chemotherapy or adjuvant chemotherapy using a regimen including anthracyclines and ifosfamide, typically spanning three treatment cycles. Due to the small size of the early metastatic lesions, as in the case we reported, it was challenging to determine whether these patients had preoperative distant metastasis. Taking this into account and in conjunction with the guidelines, we suggest that all patients diagnosed with leiomyosarcoma should be considered for a multidisciplinary treatment strategy. However, the toxic effects of these chemotherapy regimens remain a critical consideration. Early initiation of chemotherapy may have played a positive role in improving the prognosis.

## Conclusions

For leiomyosarcoma with strong concealment and high metastatic potential, multiple clinical departments, formed with vascular surgery, thoracic surgery and oncology department, should collaborate to determine the best treatment approach. Based on literature, relevant clinical research and our clinical experience, preoperative systemic evaluation conducted with the participation of thoracic surgeons is crucial for vascular leiomyosarcoma patients with lung metastasis. Radical surgical resection combined with radiotherapy can reduce local recurrence risks, particularly in patients undergoing unplanned repeat extended resection. Chemotherapy regimens should be initiated when patient’s physical conditions permit, with neoadjuvant chemotherapy being the preferred option. Regular imaging monitoring facilitates early detection and intervention of metastatic lesions, thereby improving long-term prognosis. Given the rarity of the disease, randomized controlled trials remain challenging. Future research should focus on accumulating clinical case data to explore personalized treatment approaches.

**Table 1:** (Summary of literature review regarding the GSV-LMS with metastasis cases)

Case	Author	Year	Gender/age(years)	Site	Initialsymptom	Size (mm)	Operation	Adjuvant therapy	Local recurrence	Metastasis	Postoperative follow-up (months)	Outcome
1	Szasz [26]	1969	M/68	Right thigh	NA	55	Radical resection	No	Yes	Liver/check	48	Death
2	Gross [15]	1975	M/46	Right thigh	Painless mass	50	Radical resection	Radiotherapy	No	Thyroid/subcutaneous	36	Alive
3	Stringer [17]	1977	F/36	Left thigh	Painful mass, edema	NA	Radical resection	Radiotherapy and chemotherapy	No	Lung/scalp/chest/ bone/heart	132	Death
4	Berlin [27]	1984	M/60	Right groin	Painless mass, femoral vein thrombosis	30	Extended resection	No	No	Lung/liver	1	Death
5	Dzsinch [19]	1992	F/54	NA	NA	NA	Radical resection	NA	No	Lung	9	Alive
6	Saglik [16]	1992	F/61	Right thigh	Painless mass	60	Radical resection	Chemotherapy	Yes	Lung	66	Alive
7	Reix [18]	1998	M/64	NA	NA	50	Radical resection	Chemotherapy	No	Skin/lung/brain	72	Death
8	Mammano [28]	2008	M/48	Right groin	Painless mass	60	Radical resection and femoral vein reconstruction	No	No	Lung	30	Death
9	Amato [29]	2013	M/72	Left thigh	Painless mass	60	Radical resection	Radiotherapy	No	Lung/bone	11	Death
10	Güner [30]	2020	M/34	NA	History of GSV-LMS	NA	Extended resection	NA	No	Cardiac metastasis	36	Alive
11	Ammar Atieh [12]	2024	F/63	Groin	Painless swelling	60	Extended resection	Chemotherapy	No	Lung	NA	Alive
12	Liu, Y [24]	2024	M/37	Left thigh	Painless swelling	60	Radical resection	No	No	Lung/liver	13	Alive
13	This case	2023	F/64	Right calf	superficial varicose veins with painful indurations	60	Extended resection	Radiotherapy and chemotherapy	No	Lung	36	Alive

## Data Availability

No datasets were generated or analysed during the current study.
